# Nuclear FGFR2 negatively regulates hypoxia-induced cell invasion in prostate cancer by interacting with HIF-1 and HIF-2

**DOI:** 10.1038/s41598-019-39843-6

**Published:** 2019-03-05

**Authors:** Jae Eun Lee, Seung-Hyun Shin, Hyun-Woo Shin, Yang-Sook Chun, Jong-Wan Park

**Affiliations:** 1 Department of Biomedical Science, Seoul National University College of Medicine, Seoul, Korea; 2 Department of Pharmacology, Seoul National University College of Medicine, Seoul, Korea; 3 Cancer Research Institute and Ischemic/Hypoxic Disease Institute, Seoul National University College of Medicine, Seoul, Korea

## Abstract

The fibroblast growth factor receptor 2 (FGFR2) is a membrane receptor that promotes cell proliferation and differentiation. FGFR2 is also present in the nucleus, which raises a question on a new role of FGFR2 in regulating gene expression. Hypoxia-inducible factors 1 and 2 (HIF-1 and HIF-2) are nuclear proteins that transactivate many genes essential for cancer survival and metastasis under hypoxic conditions. Here, we investigated if nuclear FGFR2 modulates the HIF-driven hypoxic response. Using the TCGA database, we found that FGFR2 downregulation is associated with poor prognosis in prostate cancer. A gene-set enrichment analysis showed that metastasis- and hypoxia-related genes are associated with a low expression of FGFR2 in prostate cancer. Thus, we tested the possibility that FGFR2 negatively regulates the hypoxia-triggered metastasis of prostate cancer. FGFR2 controls migration and invasion of prostate cancer cells under hypoxia by inhibiting the HIF-driven gene expression. FGFR2 and HIF proteins co-localize and associate in the nucleus under hypoxia. FGFR2 interacts with the transactivation domain of HIF-1α and blocks the recruitment of coactivator p300, resulting in repression of HIF target genes. Based on these results, we propose a novel function of FGFR2 as a metastasis suppressor by controlling HIF-mediated hypoxic responses.

## Introduction

Hypoxia-inducible factor 1 and 2 (HIF-1 and HIF-2), which belong to the basic helix-loop-helix (bHLH)/PER-ARNT-SIM (PAS) domain family of transcription factors, are essential for cell survival in oxygen deficiency. They are composed of two subunits; HIF-1α (or HIF-2α) and ARNT^1^. While ARNT is constitutively present in the cell, the stability of the HIF-α proteins depends on ambient oxygen tension. The de novo synthesis of HIF-1α protein is stimulated via the RAS/PI3K/AKT pathway that is activated by growth factor receptors^2^. When oxygen is present, HIF-1/2α are hydroxylated on conserved proline residues within the oxygen-dependent degradation domain by PHD1-3. This modification allows the E3 ubiquitin ligase von Hippel-Lindau (VHL) to ubiquitinate and subsequently degrade HIF-1/2α^3,4^. In addition, Factor Inhibiting HIF (FIH) hydroxylates an asparagine residue on the C-terminal transactivation domain of HIF-1/2α, which prevents the binding of the cofactors p300/CBP to HIF-1/2α, thereby inhibiting the HIF-driven transcription^5^. As these hydroxylases utilize O_2_ as a co-substrate, HIF-1/2α become stable and active under O_2_-deficient conditions. HIF-1/2α dimerize with ARNT in the nucleus, and express hypoxia-related genes essential for angiogenesis, cell movement, anaerobic metabolism, and apoptosis^6^.

The fibroblast growth factor receptor (FGFR) family belongs to the immunoglobulin superfamily and has three extracellular immunoglobulin-like domains and an intracellular tyrosine kinase domain. This family includes four different types of receptors (FGFR1-4), each of which has distinct affinities for FGF ligands^7^. Upon binding with FGF, the receptors form homodimer complexes and their kinase domains are activated. These receptors trigger the activation of their signaling cascades, such as AKT, RAS, and IP3 pathways, resulting in enhanced cell proliferation, differentiation and so on^8^. In particular, FGFR2 plays a crucial role in bone morphogenesis, so its mutations manifest abnormal bone development as shown in the craniosynostosis syndrome^9^. Due to various cell context and different isoforms, despite its main role as a growth factor receptor, whether this receptor is oncogenic or tumor suppressive is a controversial issue.

Although FGFR2 is known to be located mainly at the cell membrane as a receptor, the fact that it is also expressed in the nucleus raises a question on FGFR2 function – a new function of FGFR2 to modulate gene expressions^10^. For instance, epidermal growth factor receptor (EGFR), which is normally anchored to the plasma membrane, is also located in the nucleus, where it regulates the activity of the Cyclin D1 promoter^11^. Likewise, Macrophage Stimulating 1 Receptor (MST1R), which was alternatively named Recepteur d’origine nantais (RON), is also translocated to the nucleus upon hypoxic stimulation and binds to the c-JUN promoter in association with HIF-1α^12^. FGFR2 has been also reported to interact with the transcriptional factor Signal transducer and activator of transcription 5 (STAT5) in the nucleus and to act as a transcriptional coactivator^13^. These reports prompted us to a new hypothesis that nuclear FGFR2 acts as a co-modulator for the HIF-driven expression of hypoxia-related genes.

As FGF activates the RAS-AKT pathway to facilitate HIF-1α translation, its effect on cellular response to hypoxia was examined in several studies. Indeed, bFGF activates the HIF-1α signaling pathway under hypoxia and in turn, HIF-1α induces the expression of bFGF^14,15^. This suggests the existence of the HIF-1α-dependent bFGF autocrine loop. In addition, the crosstalk between the FGFR and HIF-1α signaling pathways has been also investigated. HIF-1α regulates the expression of FGFR3 in bladder cancer cells under hypoxia^16^. When glioblastoma cells were treated with a FGFR inhibitor SSR12819E, the stability of HIF-1α protein was decreased, suggesting that the FGFR signaling pathway boosts the hypoxic induction of HIF-1α^17^. In a view of molecular mechanism, however, the roles of FGFRs in hypoxic responses have not been intensively investigated so far.

Even though FGFR2 is considered to promote growth of cancer cells *per se*, it has been demonstrated to be downregulated in various cancers including prostate cancer^18–21^. Given these reports, we hypothesized that FGFR2 plays a negative role in cellular adaptation to tumor microenvironment. In this study, we addressed a role of FGFR2 in tumor response to hypoxic challenge. Summarizing our results, FGFR2 interacts with HIF-1α and HIF-2α, and represses their transcriptional activities in prostate cancer cells. We further demonstrated that FGFR2 negatively regulates cancer cell invasion under hypoxia and its expression is inversely correlated with prostate cancer progression.

## Results

### Downregulation of FGFR2 is associated with poor prognosis in prostate cancer

**Figure 1:**
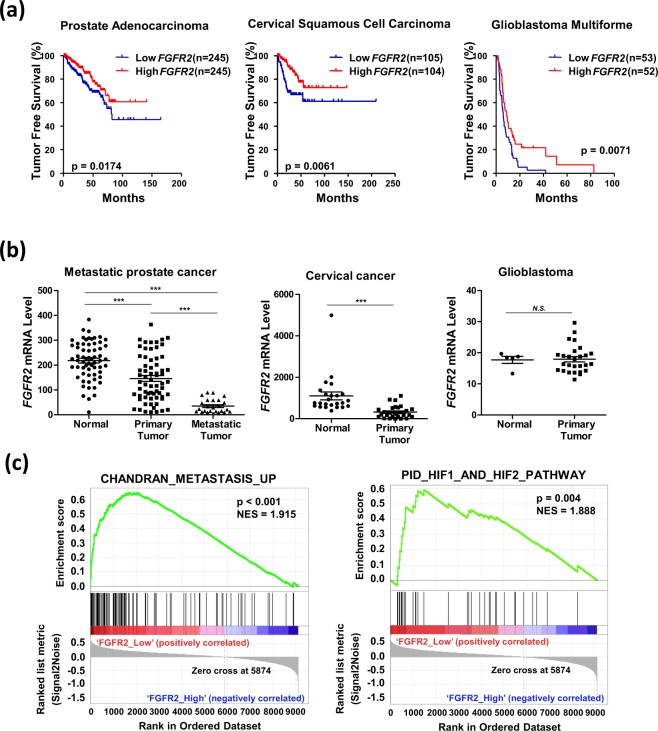
Downregulation of FGFR2 is associated with poor prognosis in prostate cancer. **(a)** Kaplan-Meier tumor free survival rates of prostate cancer, cervical squamous cell carcinoma, and glioblastoma multiforme were analyzed based on the TCGA data. *p*-value was calculated by Log-rank Test. (**b**) The relative *FGFR2* mRNA levels were analyzed with data obtained from the following GEO data sets; GSE6919 for prostate cancer, normal adjacent to tumor tissues (n = 59), primary tumor tissues (n = 66), and metastatic tumor tissues (n = 25); GSE9750 for cervical cancer, normal cervix tissues (n = 24) and cervical cancer tissue (n = 28); GSE15824 for glioblastoma, normal brain tissue (n = 5) and brain tumor tissue (n = 30). *** denotes *p* < 0.001 between the indicated groups. (**c**) GSEA enrichment plot for CHANDRAN_METASTASIS_UP gene set and PID_HIF1_AND_HIF2_PATHWAY gene set analyzed in prostate cancer.

We first examined the tumor free survival rate of 11 different types of cancer using data obtained from the TCGA database. Patients were divided into Low_*FGFR2* and High_*FGFR2* groups in respect to the median value. Informatics analyses revealed that a low expression of FGFR2 mRNA was significantly associated with poor prognosis in prostate adenocarcinoma, cervical squamous cell carcinoma, and glioblastoma multiforme (Fig. 1a, Supplementary Fig. 1). We next compared the relative expression of *FGFR2* mRNA levels in normal tissues and tumor tissues using NCBI Gene Expression Omnibus (GEO) datasets. *FGFR2* mRNA levels were reduced in tumor tissues compared to normal tissues in prostate cancer and cervical cancer, but not in glioblastoma multiforme (Fig. 1b). In prostate cancer, the *FGFR2* mRNA level was significantly lower in metastatic tumors than in either normal tissues or primary tumors. To investigate how the downregulation of FGFR2 was relevant to cancer progression, we performed Gene Set Enrichment Analyses (GSEA) to identify gene sets related to the low expression of FGFR2. Analyzing the GSEA data, we found that in prostate cancer the CHANDRAN_METASTASIS_UP (listed in Supplementary Table 2) gene set and a predefined HIF-1 and HIF-2 gene set PID_HIF1_AND_HIF2_PATHWAY (listed in Supplementary Table 3) were significantly enriched in the Low_FGFR2 *g*roup (Fig. 1c). However, these gene sets were not enriched in cervical cancer (Supplementary Fig. S2). Downregulation of FGFR2 is likely to be associated with poor prognosis in prostate cancer, but not in other types of cancers.

### FGFR2 attenuates the HIF-mediated migration and invasion of prostate cancer cells

**Figure 2:**
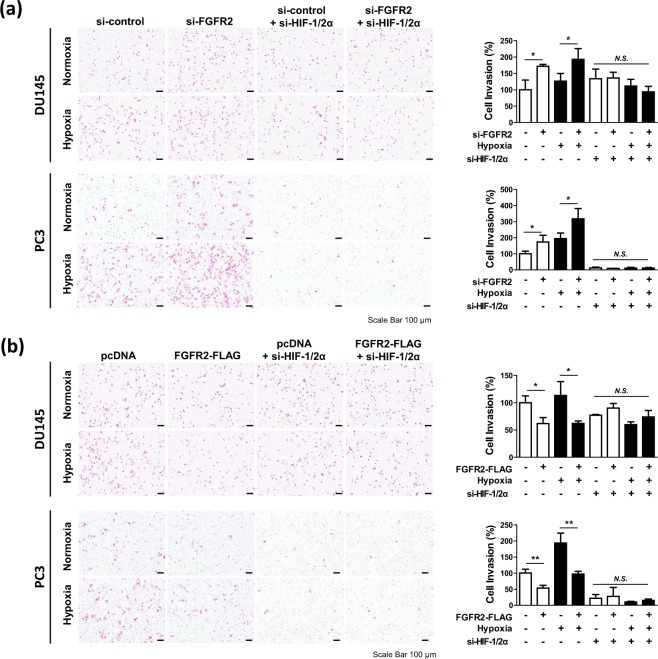
FGFR2 attenuates the HIF-induced cell invasion in prostate cancer. DU145 and PC3 cells were co-transfected with si-FGFR2 (**a**) or FGFR2-FLAG (**b**), si-HIF-1α and si-HIF-2α, and subjected to Matrigel invasion analyses in Boyden chambers. After incubated in normoxic or hypoxic conditions for 24 hours, cells on the lower surface of the Matrigel-coated membrane were stained with hematoxylin and eosin, and photographed under an optical microscope (left panel). Cells were counted and migrated cell numbers are presented as the means + SD. * and N.S. denote p < 0.05 and not significant, respectively (right panel).

Based on the GSEA results, we hypothesized that FGFR2 plays a role in prostate cancer metastasis under hypoxic conditions. We investigated the effects of FGFR2 knockdown and overexpression on migration and invasion of DU145 and PC3 cells. Knockdown of FGFR2 enhanced the migration and invasion of DU145 and PC3 cells under both normoxic and hypoxic conditions. However, this effect was abolished when HIF-1α and HIF-2α were co-silenced (Fig. 2a, Supplementary Fig. S3a). Overexpression of FGFR2 repressed cell migration and invasion, but this effect was not observed in HIF-1/2α knockdown cells (Fig. 2b, Supplementary Fig. S3b). These results suggest that FGFR2 inhibits migration and invasion of prostate cancer cells by targeting the HIF signaling pathway.

### FGFR2 weakens the HIF-driven epithelial-mesenchymal transition in prostate cancer cells

**Figure 3:**
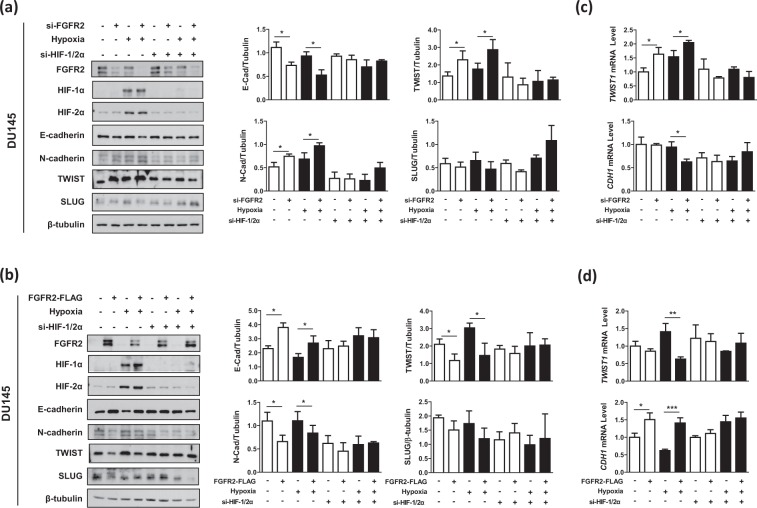
FGFR2 suppresses the HIF-mediated EMT in prostate cancer cells. DU145 cells were co-transfected with si-FGFR2 (**a**,**c**) or FGFR2-FLAG (**b**,**d**) with si-HIF-1α and si-HIF-2α, and incubated under normoxic or hypoxic conditions for 24 hours. Cells were subjected to immunoblotting with the indicated antibodies and the blots were quantified by densitometry measurements with ImageJ (right panels). The mRNA levels of *CDH1* and *TWIST* or *SNAI2* were quantified by RT-qPCR. All experiments were performed three times and results are expressed as bars representing the means + SD. * and ** denotes *p* < 0.05 and *p* < 0.01 between the indicated groups, respectively.

We next investigated whether FGFR2 inhibits the HIF-induced epithelial-mesenchymal transition (EMT). The cellular levels of an epithelial marker E-cadherin and mesenchymal markers N-cadherin, TWIST and SLUG were measured in DU145 and PC3 cells. Of the transcription factors that repress E-cadherin^22^, TWIST and SLUG were predominantly expressed in DU145 cells and PC3 cells, respectively. Knockdown of FGFR2 reduced E-cadherin expression but enhanced N-cadherin expression in both cells. It also upregulated TWIST in DU145 cells or SLUG in PC3 cells (Fig. 3a, Supplementary Fig. 4a). Overexpression of FGFR2 showed the opposite results (Fig. 3b, Supplementary Fig. 4b). Both effects of FGFR2 knockdown and overexpression were nearly abolished by co-silencing of HIF-1α and HIF-2α. In RT-qPCR analyses, when FGFR2 was silenced, the *TWIST1* and *SNAI2* mRNAs increased in DU145 cells and PC3 cells, respectively, while the *CDH1* mRNA decreased in both cell lines (Fig. 3c, Supplementary Fig. 4c). Ectopic expression of FGFR2 showed the opposite results (Fig. 3d, Supplementary Fig. 4d), which was attenuated by HIF-1/2α co-silencing.

### FGFR2 represses the HIF-driven transcription of target genes

**Figure 4:**
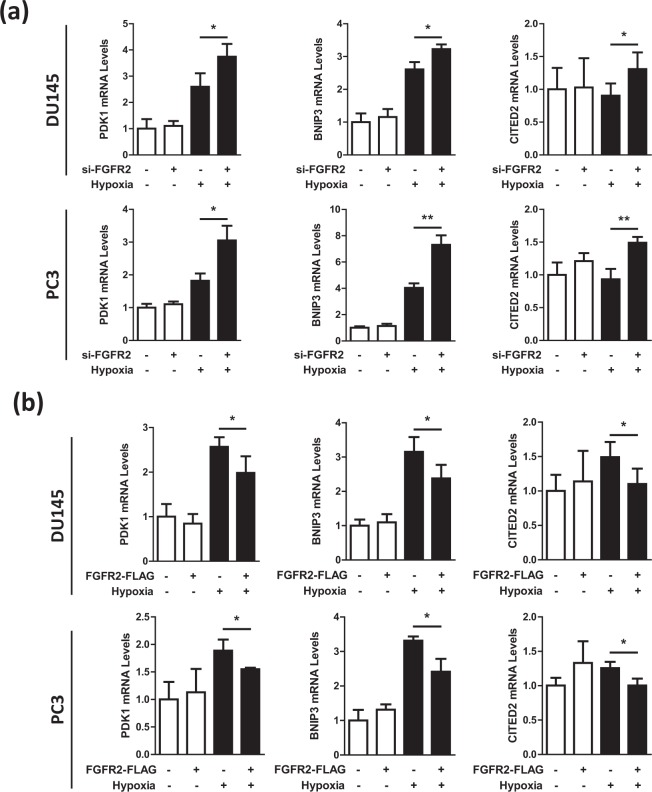
FGFR2 inhibits the expression of HIF target genes under hypoxia. DU145 and PC3 cells, which had been co-transfected with si-FGFR2 (**a**) or FGFR2-FLAG (**b**) were incubated in normoxic or hypoxic conditions for 24 hours. Cells were lysed for the extraction of RNAs, which were subjected to RT-qPCR for analyzing the mRNA levels of HIF-1/2 target genes. Relative mRNA levels were presented as the means and SD (n = 3). * and ** denote p < 0.05 and p < 0.01, respectively.

**Figure 5:**
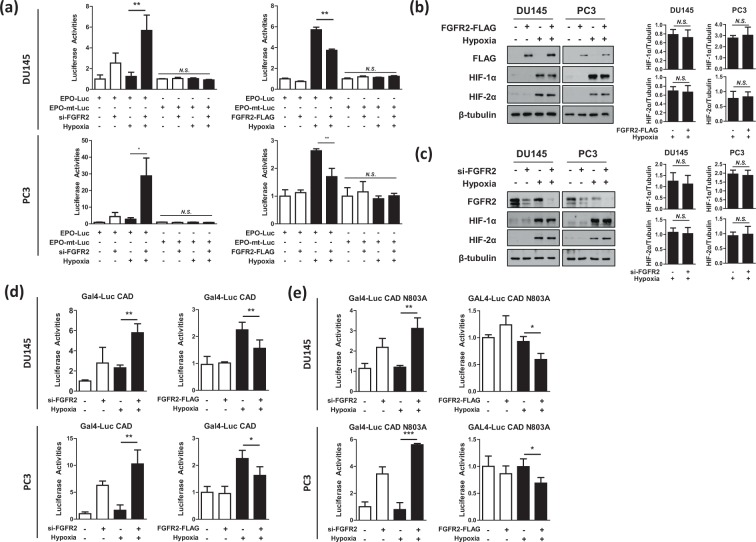
FGFR2 inhibits HIF-1 by repressing the CAD activity. (**a**) DU145 and PC3 cells were co-transfected with EPO enhancer-Luc plasmid, CMV-β-gal plasmid, and si-FGFR2 (or FGFR2-FLAG), and incubated under normoxic or hypoxic conditions for 16 hours. Luciferase activities were determined and presented as relative values (the means + SD, n = 3) versus the normoxic control. * and ** denotes *p* < 0.05 and *p* < 0.01 between the indicated groups, respectively. (**b**,**c**) DU145 and PC3 cells, which had been transfected with si-FGFR2 or FGFR2-FLAG, were incubated under normoxic or hypoxic conditions for 24 hours and subjected to immunoblotting with the indicated antibodies. HIF-1α, and HIF-2α blots were quantified by densitometry measurements with ImageJ (right panel). (**d**,**e**) DU145 and PC3 cells were co-transfected with Gal4_promoter-Luc reporter, Gal4_DBD-HIF-1α_CAD (or CAD N803A), and si-FGFR2 (or FGFR2-FLAG). Cells were incubated in normoxia or hypoxia for 16 hours and lysed to measure luciferase activities. Each bar represents the mean + SD (n = 3). * and N.S. denote *p* < 0.05 and *p* > 0.05 between the indicated groups, respectively.

We next examined whether the HIF-driven gene expression was regulated by FGFR2. The mRNA levels of HIF-1 (*PDK1*, *BNIP3*) and HIF-2 (*CITED2*) target genes were evaluated by RT-qPCR in DU145 and PC3 cells transfected with si-FGFR2 or FGFR2-FLAG plasmid. Results showed that the mRNA expressions of HIF-1/2 downstream genes were increased by FGFR2 knockdown (Fig. 4a), but decreased by FGFR2 overexpression (Fig. 4b). To further evaluate the role of FGFR2 in the HIF-mediated transcription, the EPO-enhancer luciferase reporter system, which contains the hypoxia response element (HRE) in the erythropoietin enhancer region, was used. The transcriptional activity of HIF was augmented by FGFR2 knockdown but reduced by FGFR2 overexpression (Fig. 5a). A reporter mutated at the HRE was also tested to verify that the luciferase activity depends on HIF. As the activity of mutated reporter was not affected by FGFR2 expression, FGFR2 is likely to inhibit the reporter activity in a HIF-specific manner.

### FGFR2 control of HIF-mediated hypoxic responses is independent of a FGFR2 ligand

Ligands of FGFR2 have been reported to regulate cell migration^23,24^. Thus, we treated DU145 and PC3 cells with a FGFR2 ligand FGF10, to explore its effect on cellular behavior in hypoxia. FGF10 increased the migration of cancer cells in both normoxic and hypoxic conditions (Supplementary Fig. 5a), and also changed the levels of EMT markers (Supplementary Fig. 5b). We further assessed the effect of FGF10 on HIF-mediated transcription, and found that FGF10 did not affect the mRNA levels of HIF target genes (Supplementary Fig. 6a). Therefore, it is suggested that the FGF10-induced cell migration occurs independently of the FGFR2 control of HIF-driven hypoxic responses.

### FGFR2 inactivates the transactivation domain of HIF-1α

We next addressed the mechanism by which FGFR2 inhibits HIF. The knockdown or overexpression of FGFR2 did not affect the protein expression levels of HIF-1α and HIF-2α (Fig. 5b,c). From these results, we tested the possibility that FGFR2 functionally inhibits HIF. As the C-terminal transactivation domain (CAD) is responsible for hypoxia-induced activation of HIF-1α, we analyzed the activity of HIF-1α CAD in DU145 and PC3 cells using the Gal4 reporter system. The activity of CAD was notably enhanced by FGFR2 knockdown, but reduced by FGFR2 overexpression (Fig. 5d). The CAD activity is oxygen-dependently regulated through the FIH hydroxylation of the N803 residue^5^. To examine whether FGFR2 affects such an action of FIH, we measured the activity of mutated CAD N803A, which is not regulated by FIH. As the activity of CAD N803A is constitutively active regardless of oxygen level, it is reasonable that its activity did not increase under hypoxia. However, FGFR2 still repressed the activity of CAD N803A (Fig. 5e). These results indicate that FGFR2 inhibits the transcriptional activity HIF-1α irrespective of FIH.

### FGFR2 interacts with both HIF-1α and HIF-2α

**Figure 6:**
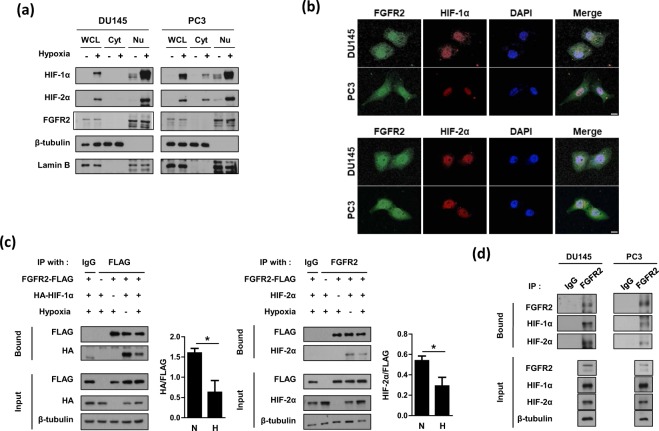
FGFR2 interacts with HIF-1α and HIF-2α in the nucleus. (**a**) DU145 and PC3 cells were incubated in hypoxia for 8 hours and whole cell lysates (WCL) were fractionated into cytosolic (Cyt) and nuclear (Nu) compartments. These fractions were subjected to immunoblotting with the indicated antibodies. (**b**) Representative immunofluorescence images. DU145 and PC3 cells were grown on coverslips and subjected to hypoxia for 8 hours. Samples were then fixed with 4% paraformaldehyde, and stained with the indicated antibodies. All samples were stained with DAPI to visualize nuclei. The scale bar represents 10 μm. (**c**) HEK293T were co-transfected with FGFR2-FLAG and HA-HIF-1α (or HIF-2α), and incubated under normoxic or hypoxic conditions for 8 hours. Cell lysates were subjected to immunoprecipitation with IgG, anti-FLAG, or anti-FGFR2, and the bound proteins were immunoblotted with the indicated antibodies. The blots of bound HA and bound HIF-2α were quantified using ImageJ (right panel). * denotes *p* < 0.05 between the indicated groups. (**d**) DU145 and PC3 cells were incubated under normoxic or hypoxic conditions for 8 hours. Cell lysates were subjected to immunoprecipitation with IgG or anti-FGFR2, and the bound proteins were immunoblotted with the indicated antibodies.

FGFR2 is known to be anchored to the plasma membrane^25^, but HIF-1/2α are localized in the nucleus^26^. Recent studies have demonstrated that FGFR2 exists in the nucleus, where it interacts with the transcription factor STAT5^13,27^. Therefore, we next tested the possibility of interaction between FGFR2 and HIF-1/2α in the nucleus. Immunoblots of cytosolic and nuclear fractions from DU145 and PC3 cells showed that a substantial amount of FGFR2 was detected in the nuclear fraction (Fig. 6a). In contrast, the amount of nuclear FGFR2 was negligible in HEK293 and DLD-1 and marginal in MCF7 (Supplementary Fig. 7). Therefore, the nuclear localization of FGFR2 occurs depending on cell context and it seems to be activated especially in prostate cancer cells. Using immunofluorescence analysis, we further demonstrated that FGFR2 and HIF-1/2α co-localized in the nuclei of DU145 and PC3 cells (Fig. 6b). We continued to examine the interaction between FGFR2 and HIF-1/2α by performing co-immunoprecipitation. When ectopically expressed, HIF-1α and HIF-2α proteins co-immunoprecipitated with FGFR2 (Fig. 6c). Interestingly, the protein interactions were stronger in normoxia than in hypoxia. Endogenous interaction of FGFR2 with HIF-1α or HIF-2α in hypoxic conditions was also confirmed in DU145 and PC3 cells (Fig. 6d).

### FGFR2 interferes with the p300 recruitment by HIF-1α CAD

**Figure 7:**
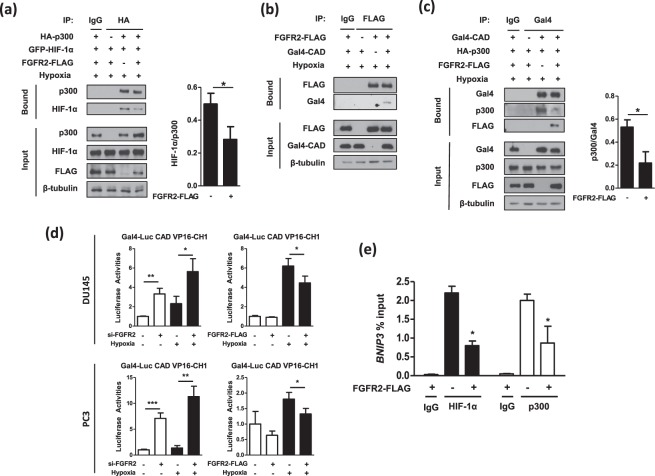
FGFR2 disturbs the interaction between HIF-1α_CAD and p300. (**a**) HEK293T cells were co-transfected with HA-p300, GFP-HIF-1α, and FGFR2-FLAG and incubated in hypoxia for 8 hours. Interaction between p300 and HIF-1α was examined by immunoprecipitation with anti-HA affinity gel, and the co-precipitated complexes were subjected to immunoblotting. The blots of bound HIF-1α were quantified using ImageJ (right panel). (**b**) HEK293T cells were co-transfected with FGFR2-FLAG plasmid and Gal4-CAD plasmid and incubated in hypoxic conditions for 8 hours. Cell extracts were immunoprecipitated with anti-FLAG affinity gel, and co-precipitated Gal4-CAD was examined with anti-Gal4 antibody. (**c**) Gal4-CAD, HA-p300, and FGFR2-FLAG was overexpressed in HEK293T cells and incubated in hypoxic conditions for 8 hours. Cell extracts were immunoprecipitated with anti-Gal4, and co-precipitated proteins were examined with the indicated antibodies through immunoblotting. The bands of bound p300 were quantified by densitometry measurements with ImageJ. (**d**) DU145 and PC3 cells were co-transfected with si-FGFR2 (or FGFR2-FLAG), Gal4-Luc reporter, Gal4-CAD, and VP16-CH1 plasmids. Cells were incubated in normoxic or hypoxic conditions for 16 hours and further lysed to determine luciferase activities. (**e**) HIF-1α or p300 binding to the BNIP3 promoter region was detected by ChIP-qPCR using non-immunized serum (IgG), anti-HIF-1α or anti-p300 antiserum in PC3 cells. Bars represent the means + SD (n = 3) and * denotes *p* < 0.05 between the indicated groups.

To identify the mechanism by which FGFR2 inhibits the HIF activity through protein interaction, we investigated the p300 binding to HIF-1α because p300 plays a critical role in HIF-driven transcription^6^. We performed the immunoprecipitation analysis using HA-p300 and GFP-HIF-1α. Subsequently, the interaction between p300 and HIF-1α was attenuated by FGFR2 overexpression (Fig. 7a). Since binding competitors usually bind to the common motif in their partner, we tested the possibility that FGFR2 interacts with the CAD that is the binding motif for p300. Immunoprecipitation and immunoblotting analyses revealed that FGFR2 interacted with the HIF-1α CAD (Fig. 7b). When Gal4-CAD was immunoprecipitated, FGFR2 overexpression reinforced the interaction between FGFR2 and CAD and simultaneously dissociated p300 from CAD (Fig. 7c). To confirm this binding competition, we adopted the mammalian two-hybrid system using Gal4 (DBD)-HIF-1α_CAD and p300_CH1-VP16 (TAD). The CH1 domain of p300 is the specific docking site for CAD interaction. In both DU145 and PC3 cells, the reporter activities were markedly elevated by FGFR2 knockdown, but diminished by FGFR2 overexpression (Fig. 7d). We further performed ChIP-qPCR to explore the effect of FGFR2 on the binding of HIF-1α or p300 with the *BNIP* promoter in PC3 cells. Overexpression of FGFR2 significantly decreased the recruitment of HIF-1α and p300 to the *BNIP* promoter (Fig. 7e). Taken together, FGFR2 binds to HIF-1α and by doing so interferes with HIF-1α targeting to downstream genes and its interaction with p300.

## Discussion

In case of regional prostate cancer, the 5-year survival rate is almost 100%. However, the survival rate dramatically drops when the tumor spreads out beyond the prostate, implying metastasis is the main reason accounting for prostate cancer mortality^28^. Therefore, preventing metastasis in prostate cancer is crucial for patient survival. As the HIF-mediated hypoxic signaling makes tumors more aggressive and metastatic^29^, anticancer strategies to target HIF have been tried. Several reports have suggested the possibility of FGFR engaging in the hypoxia pathway. Even so, most studies have focused on the involvement of the receptor-mediated signal transduction in hypoxic responses. In this study, we identified FGFR2 as a co-modulator of the HIF-driven transcription in the nucleus. It was also demonstrated that FGFR2 lowers the metastatic potential of prostate cancer cells under hypoxia by inhibiting HIF.

Many studies have identified different regulators that modulate the transcriptional activity of HIF^30^. FIH, one of the most representative regulators of HIF activity, has been revealed to hydroxylate an asparagine residue on the CAD of HIF-1α, preventing the interaction between HIF-1α and CBP/p300^5^. Casein kinase I phosphorylates Ser247 of HIF-1α, and this modification interferes the dimerization of HIF-1α and HIF-1β, leading to a decrease of HIF transcriptional activity^31^. On the other hand, casein kinase II was reported to phosphorylate Thr796 of HIF-1α, and this phosphorylation blocks hydroxylation by FIH, resulting in activation of HIF^32^. p42/p44 MAPK was demonstrated to induce HIF-1α phosphorylation and increase its transcriptional activity^33^, and protein kinase A also has been identified to regulate HIF-1α protein stability and transcriptional activity^34^. S-nitrosylation of the thiol group of Cys800 of HIF-1α and SUMOylation of HIF-1α has also been reported to modify the transcriptional activity of HIF-1, but still remain controversial^35–38^. These findings also suggest the possibility of a new post-translational modification of HIF-α by FGFR2. Using the NetPhos program (http://www.cbs.dtu.dk/services/NetPhos/), the conserved residues Tyr798 of HIF-1α CAD and Tyr842 of HIF-2α CAD are predicted to be potentially phosphorylated by receptor tyrosine kinases. With previous studies showing that post-translational modifications of HIF-1/2 α affect its transcriptional function, it is possible that FGFR2 phosphorylates HIF-1α and by doing so disturbs the interaction between HIF-1α and p300. Otherwise, it is also possible that the FGFR2 binding to CAD physically blocks the access of p300 to CAD. The mechanism by which FGFR2 disturbs the p300 binding to CAD remains to be investigated.

In reference to reports showing that membrane receptors act as co-modulators for transcription factors in the nucleus, we investigated whether FGFR2 would interact with HIF-1α and HIF-2α. Surprisingly, the receptor interacted with both transcription factors, and these interactions were observed to decrease in hypoxia versus in normoxia. The O_2_ tension-dependent change in this interaction implies that FGFR2 may function as checkpoint in HIF-1/2α transcriptional activities. The stronger interactions in normoxia seem to ensure that HIF-1/2α have no transcriptional activity unnecessary in non-hypoxic conditions. The weaker interactions between FGFR2 and HIF-1/2α during hypoxia may allow HIF-1/2α to be highly active in expressing targeted genes.

Because activated FGFR signaling leads to increased angiogenesis, survival, and proliferation, many efforts have been made to target the signaling of FGFR in cancer therapy^39^. Among the FGFR family members, FGFR1 has been reported to promote the progression of prostate cancer^40–42^. In contrast to the consistent reports of FGFR1 as an oncogene, the connection between FGFR2 and prostate cancer progression still remains controversial. Although one might expect that growth factor receptors to be overexpressed in cancer, several studies have reported that FGFR2 mRNA expression was downregulated in cancer^18,41–44^, which is also consistent to our results. In regard of cancer progression, therefore, the FGFR family members seem to have distinct roles. In conclusion, we identified FGFR2 as a new regulator of HIF transcriptional activity. FGFR2 interacts with HIF-1/2α in the nucleus and by doing so inhibits the recruitment of p300 by HIF-1/2α. This interruption by FGFR2 resulted in a decrease of HIF transcriptional activity. Furthermore, FGFR2 was found to inhibit the HIF-induced migration and invasion of prostate cancer cells, providing an explanation why the mRNA levels of FGFR2 were downregulated in tumor and metastatic tissues. Based on these results, we propose that FGFR2 plays a role in regulating hypoxia responses as a checkpoint in the activation of HIF transactivation domain.

## Methods

### Reagents and antibodies

Culture media were purchased from Invitrogen (Carlsbad, CA), and fetal bovine serum from Sigma-Aldrich (St. Louis, MO). Antibodies against FGFR2, β-tubulin, p300, Gal4 (DBD), Lamin B, N-Cadherin, TWIST, and SLUG were purchased from Santa Cruz Biotechnology (Santa Cruz, CA); anti-E-cadherin from Cell Signaling (Danvers, MA); anti-HIF-2α from Novus Biologicals (Littleton, CO); anti-FLAG from Sigma-Aldrich (St. Louis, MO); anti-HA from Roche Diagnostics (Mannheim, Germany). Anti-HIF-1α antibody was raised in rabbits against human HIF-1α, as previously described^45^. The recombinant FGF10 protein was purchased from Peprotech (Rocky Hill, NJ).

### Preparation of siRNAs and plasmids

Hemagglutinin (HA)-tagged HIF-1α, green fluorescence protein (GFP)-tagged HIF-1α, HA-tagged p300, EPO enhancer-luciferase reporter, Gal4-CAD (aa. 776–826 of HIF-1α, Gal4-CAD N803A mutant, VP16-p300 CH1 plasmids were constructed, as previously described^46^. Full-length FGFR2-3XFLAG plasmid was a kind gift from Dr. Hyun-Mo Ryoo (Seoul National University, South Korea). The sequences of siRNAs used are; 5′- AAGCAGUGGGAAUCGAUAAAGACAA -3′ for FGFR2 (NM_022970), 5′- GAAGGAACCUGAUGCUUUAACUUTG -3′ for HIF-1α (NM_001530), 5′- GGGUUACUGACGUGUAAAUGCUGGT -3′ for HIF-2α (NM_001430), 5′- UUGAGCAAUUCACGUUCAUTT -3′ for control.

### Cell culture and transfection

Human embryonic kidney (HEK293 and HEK293T), human prostate cancer (DU145, PC3) cell lines were obtained from ATCC. HEK293 and HEK293T were cultured in DMEM, DU145 and PC3 in RPMI, with 10% heat-inactivated fetal bovine serum (FBS), and penicillin-streptomycin in a 5% CO_2_ humidified atmosphere at 37 °C. Mycoplasma contamination was routinely checked when cell growth or shape was changed. The oxygen tension in the incubator (Vision Sci, Seoul, Korea) was 20% for normoxia or 1% for hypoxia. For transient transfection, cells seeded at an approximately 50% confluency were transfected with plasmids or siRNAs using calcium phosphate, Lipofectamine 3000 reagent (Invitrogen), or Lipofectamine RNAiMAX reagent (Invitrogen).

### Reporter and mammalian two-hybrid assays

To examine HIF-1 and HIF-2 activities, HEK293, DU145 and PC3 cells were co-transfected with 1 µg of EPO-enhancer luciferase, 1 µg of CMV-β-gal plasmid, 1 µg of FGFR2-FLAG plasmid or 40 mM siRNA targeting FGFR2 using calcium phosphate or Lipofectamine 3000 reagent. To examine HIF-1α CAD activity, DU145 and PC3 cells were co-transfected with 100 ng of Gal4-promoter luciferase, 100 ng of Gal4-CAD, 1 µg of CMV-β-gal plasmid, 1 µg of FGFR2-FLAG plasmid or 40 mM siRNA targeting FGFR2 using Lipofectamine 3000 reagent. To examine the interaction between CAD and p300, DU145 and PC3 cells were co-transfected with 100 ng of Gal4-promoter luciferase, 100 ng of Gal4-CAD, 500 ng of VP16-CH1 plasmid, 1 µg of CMV-β-gal plasmid, 1 µg of FGFR2-FLAG plasmid or 40 mM of siRNA targeting FGFR2 using Lipofectamine 3000 reagent. The final concentrations of DNA or siRNA were adjusted with pcDNA or control siRNA. After being stabilized for 48 hours, the cells were incubated under normoxic or hypoxic conditions for 16 hours, and then lysed and assayed for luciferase activities while β-gal activities were determined to normalize transfection efficiency.

### Immunoblotting and immunoprecipitation

Cell lysates were separated on sodium dodecylsulfate (SDS)-polyacrylamide (7–15%) gels, and transferred on to Immobilion-P membranes (Millipore; Bedford, MA). Membranes were blocked with Tris-buffered saline solution containing 5% skim milk and 0.1% Tween-20 for 1 hour and incubated with a primary antibody overnight at 4 °C. The membranes were incubated with a horseradish peroxidase-conjugated secondary antibody for 1 hour and visualized using the ECL Plus kit (Amersham Biosciences; Piscataway, NJ). To analyze protein interactions, cell lysates were incubated with anti-FGFR2 antibodies overnight at 4 °C, and the immune complexes were precipitated using protein A/G beads (Santa Cruz, CA). Otherwise, cell lysates were incubated with EZview Red anti-HA or anti-FLAG affinity gel (Sigma-Aldrich) overnight at 4 °C. Precipitated immunocomplexes were eluded with a denaturing 2xSDS sample buffer and subjected to immunoblotting. Immunoblots were quantified using the ImageJ program (NIH, Bethesda, Maryland).

### Immunofluorescence

DU145 and PC3 cells grown on cover glass slides were fixed with 4% paraformaldehyde for 10 minutes, and then permeablized with 0.25% Triton X-100 for 20 minutes. Cells were blocked with 3% BSA for 1 hour and incubated with a primary antibody at 4 °C in the dark overnight. Alexa Flour® 594 anti-rabbit or Alexa Flour® 488 anti-mouse solution was added and cells were incubated in the dark for 1 hour. The nuclei were stained with 4′, 6-diamidino-2-phenylindole (DAPI) for 20 min, and fluorescence images were photographed using confocal microscopy.

### Bioinformatics analysis

Gene set enrichment analysis (GSEA) was performed using GSE6919 data set, and a formatted GCT file was used as input for the GSEA algorithm v2.0 (available from: http://www.broadinstitute.org/gsea). mRNA data sets were obtained from The Cancer Genome Atlas (TCGA), and the values of the 1363_at probe were used as criteria standard for FGFR2 low expression and FGFR2 high expression group.

### Migration and invasion assays

DU145 and PC3 cells were cultured in cell culture inserts with an 8 µm pore size membrane purchased from Corning Life Science (Acton, MA). For cell migration analysis, DU145 and PC3 cells were seeded in the upper transwell chambers containing serum-free media while the lower chambers contained the medium supplemented with 10% FBS to induce cell migration. For cell invasion analysis, the membrane was coated with 0.5 mg/ml of Matrigel (Corning Life Science, Acton, MA). After incubation in normoxic or hypoxic conditions for 24 hours, the cells were fixed with 4% paraformaldehyde and stained with hematoxylin and eosin (Sigma-Aldrich). Cells on the upper side of the interface membrane were removed with a cotton swab, and migrated or invaded cells on the lower surface of the membrane were counted under an optical microscope.

### Subcellular fractionation

Cells were centrifuged at 1000 × *g* for 5 min at 4 °C, and gently resuspended in a hypotonic solution containing 20 mM Tris/HCl (pH 7.8), 1.5 mM MgCl_2_, 10 mM KCl, 0.2 mM EDTA, 0.5% NP-40, 0.5 mM dithiotheritol, and 0.5 mM PMSF. After letting the cells to swell on ice for 10 minutes, the cell lystates were centrifuged at 3000 × *g* for 10 min at 4 °C, and the supernatant was collected as the cytosolic fraction. Hypertonic solution containing 20 mM Tris/HCl (pH 7.8), 5% glycerol, 1.5 mM MgCl_2_, 400 mM NaCl, 0.2 mM EDTA, 0.5 mM dithiotheritol, and 0.5 mM PMSF was added to the pellet, and vortexed intermittently on ice for 30 min. The suspension was spun down at 13000 × *g* for 20 min at 4 °C, and the supernatant was collected as the nuclear fraction.

### Quantitative RT-PCR

Total RNA was isolated with TRIZOL reagent (Invitrogen; Carlsbad, CA), and cDNA was synthesized in a reaction mixture containing MMLV Reverse Transcriptase (Promega; Madison, WI), RNase inhibitor, random primers, dNTP and 0.1% BSA for 1 hour at 42 °C. Quantitative real-time PCR was performed in triplicates on 96-well optical plates using qPCR Mastermix (Enzynomics; Daejeon, South Korea), and fluorescence was detected by CFX ConnectTM System (Bio-Rad). PCR conditions were 40 cycles of 95 °C for 10 sec, 53 °C for 15 sec, and 72 °C for 20 sec. Data were analyzed with the CFX Manager Software (Bio-Rad), and the mRNA values of targeted genes were normalized to GAPDH expression. The sequences of PCR primers are summarized in Supplementary Table 1.

### Chromatin immunoprecipitation

Cells were fixed with 1% formaldehyde for 10 min at 37 °C and then treated with 150 mM glycine. The fixed cells were lysed with 0.5% NP-40 and centrifuged at 1000 × *g* for 5 min at 4 °C to collect the crude nuclear fraction. The nuclear pellet was suspended in 1% SDS and sonicated. Chromatin complexes were reacted with IgG, anti-HIF-1α or anti-p300 antibody overnight at 4 °C, and precipitated with protein A/G beads for 4 hours at 4 °C. After the beads were sequentially washed with low and high salt TE buffers, the immunoprecipitates were eluted at 65 °C. DNAs were isolated by phenol-chloroform-isoamyl alcohol (25:24:1) and precipitated using ethanol and glycogen overnight at −70 °C. The extracted DNA was resolved in nuclease-free water and analyzed by real-time PCR (95/60/72 °C, 30 sec at each phase).

### Statistics

All data was analyzed with the Microsoft Excel 2011 or the GraphPad Prism 7.0 software. Results were represented as the means and standard deviation (SD), and significance within the data was determined by the unpaired, two-sided Student *t*-test. Tumor free survival was examined with the Kaplan-Meier method and compared with the log-rank test. All statistical significances were considered when *p* values were under 0.05.

## Supplementary information

Supplementary Information
