# Supplementary material for: Nuclear FGFR2 negatively regulates hypoxia-induced cell invasion in prostate cancer by interacting with HIF-1 and HIF-2
Source: Sci Rep. 2019 Mar 5;9:3480. doi: 10.1038/s41598-019-39843-6 (PMC6401139; doi:10.1038/s41598-019-39843-6)
Supplement: Supplementary file 1 — Supplementary Information [file 41598_2019_39843_MOESM1_ESM.pdf]

Nuclear FGFR2 negatively regulates hypoxia-induced cell invasion in prostate cancer by interacting with HIF-1 and HIF-2

Jae Eun Lee, Seung-Hyun Shin, Hyun-Woo Shin, Yang-Sook Chun,

and Jong-Wan Park

Supplementary Information

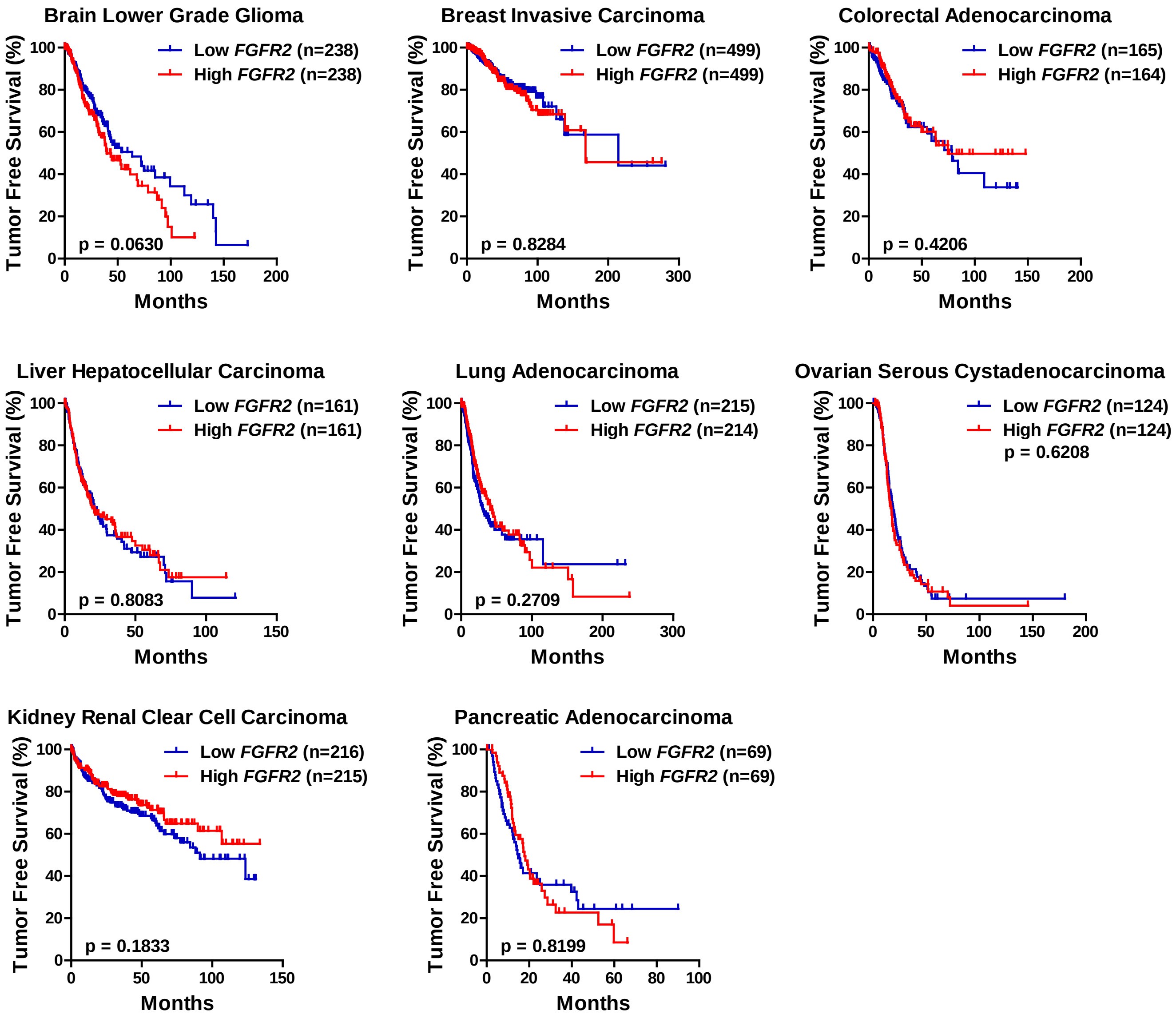

Supplementary Figure 1. Relation between FGFR2 expression and tumor-free survival in various cancers. Kaplan-Meier tumor free survival rate of brain lower grade glioma, breast invasive carcinoma, colorectal adenocarcinoma, kidney renal clear cell carcinoma liver hepatocellular carcinoma, lung adenocarcinoma, ovarian serous cystadenocarcinoma, and pancreatic adenocarcinoma was analyzed with data obtained from TCGA. p-value was calculated by Log-rank Test.

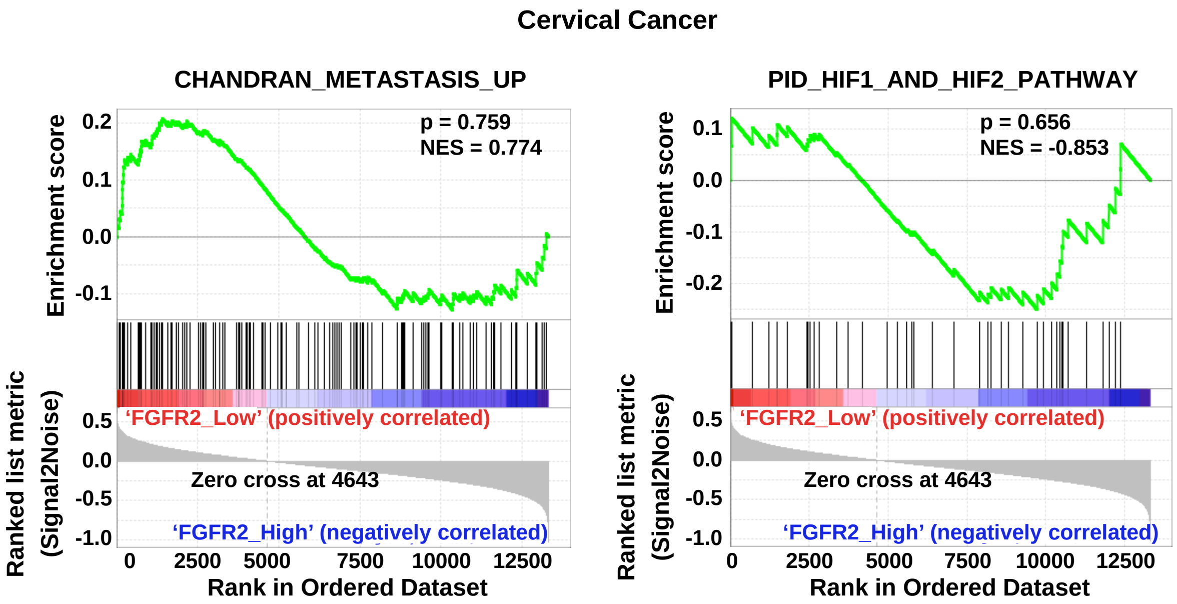

Supplementary Figure 2. GSEA enrichment plot for CHANDRAN_METASTASIS_UP gene set and PID_HIF1_AND_HIF2_PATHWAY gene set analyzed in cervical cancer.

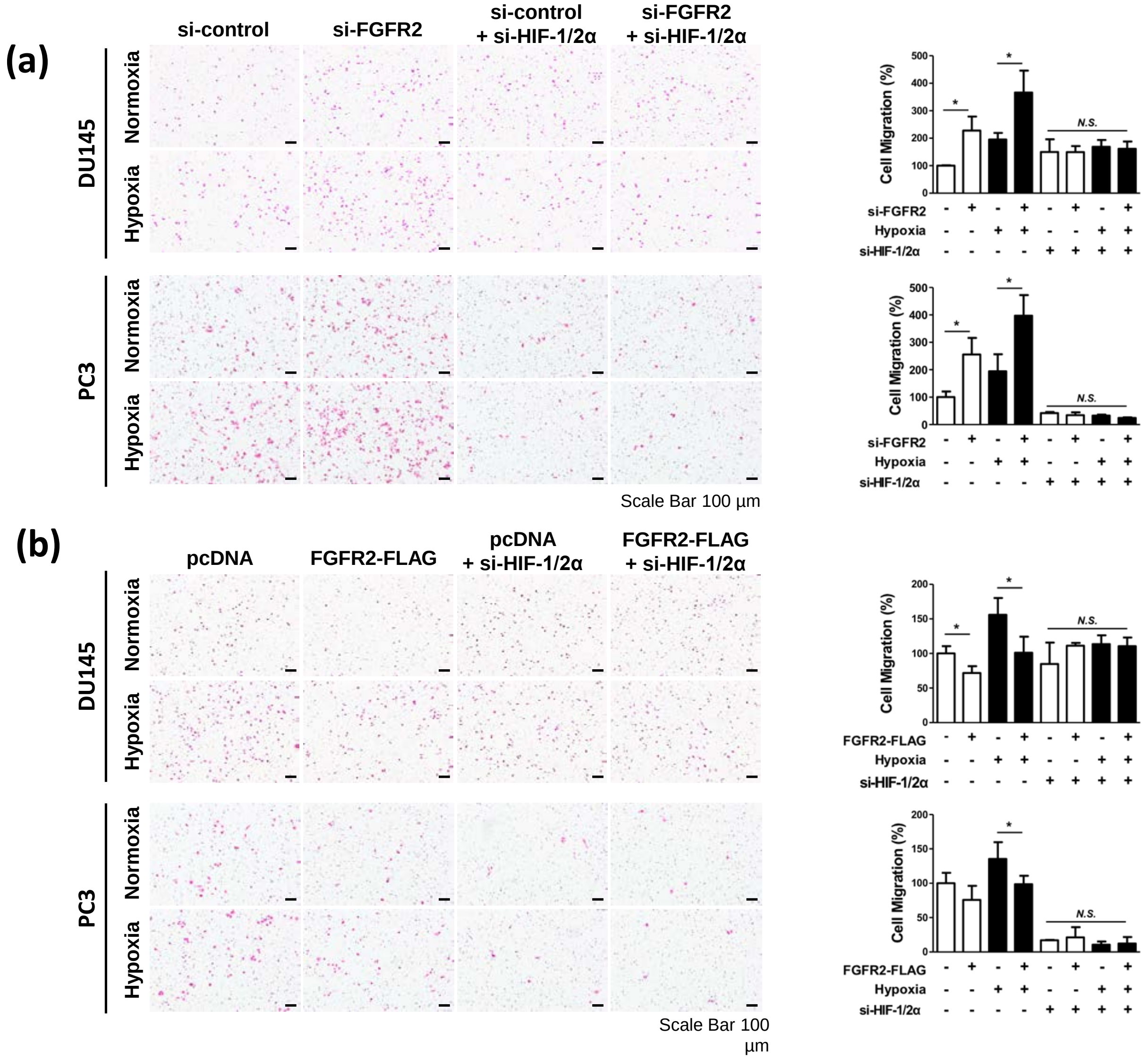

Supplementary Figure 3. FGFR2 suppresses the hypoxia-induced migration of prostate cancer cells. DU145 and PC3 cells were co-transfected with si-FGFR2 (a) or FGFR2-FLAG (b), si-HIF-1α and si-HIF-2α, and subjected to cell migration analyses. After incubated in normoxic or hypoxic conditions for 24 hours, cells on the lower surface of the membrane were stained with hematoxylin and eosin, and photographed under an optical microscope (left panel). Cells were counted and migrated cell numbers are presented as the means + SD. * and N.S. denote $p < 0.05$ and not significant, respectively (right panel).

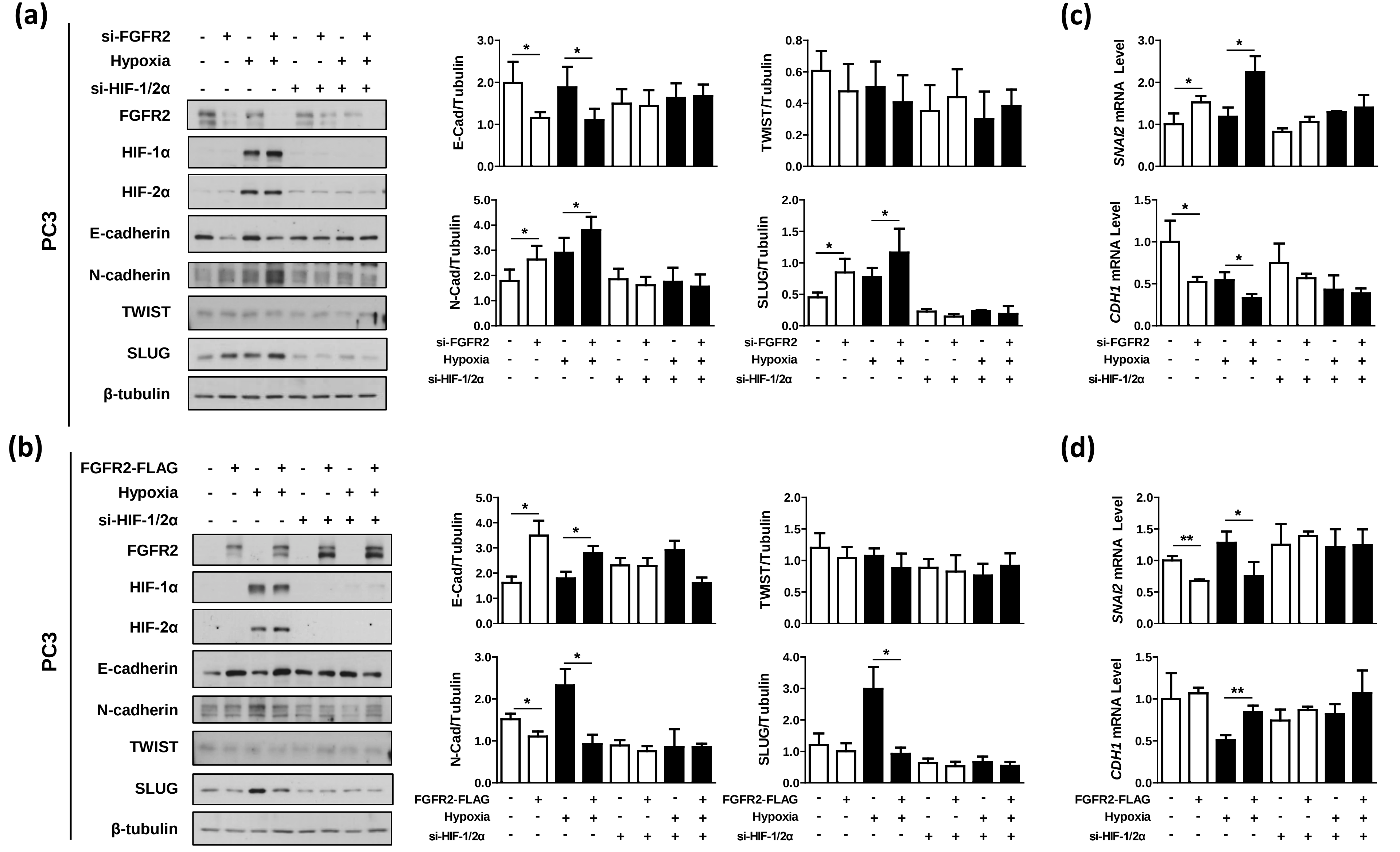

Supplementary Figure 4. FGFR2 suppresses the HIF-mediated EMT in prostate cancer cells. PC3 cells were co-transfected with siFGFR2 (a), (b) or FGFR2-FLAG (c), (d) with si-HIF-1α and si-HIF-2α, and incubated under normoxic or hypoxic conditions for 24 hours. Cells were subjected to immunoblotting with the indicated antibodies. The bands were quantified by densitometry measurements with ImageJ , and the mRNA levels of CDH1 and SNAI2 were quantified by RT-qPCR. All experiments were performed three times and results are expressed as bars representing the means + SD. * and ** denotes $p < 0.05$ and $p < 0.01$ between the indicated groups, respectively.

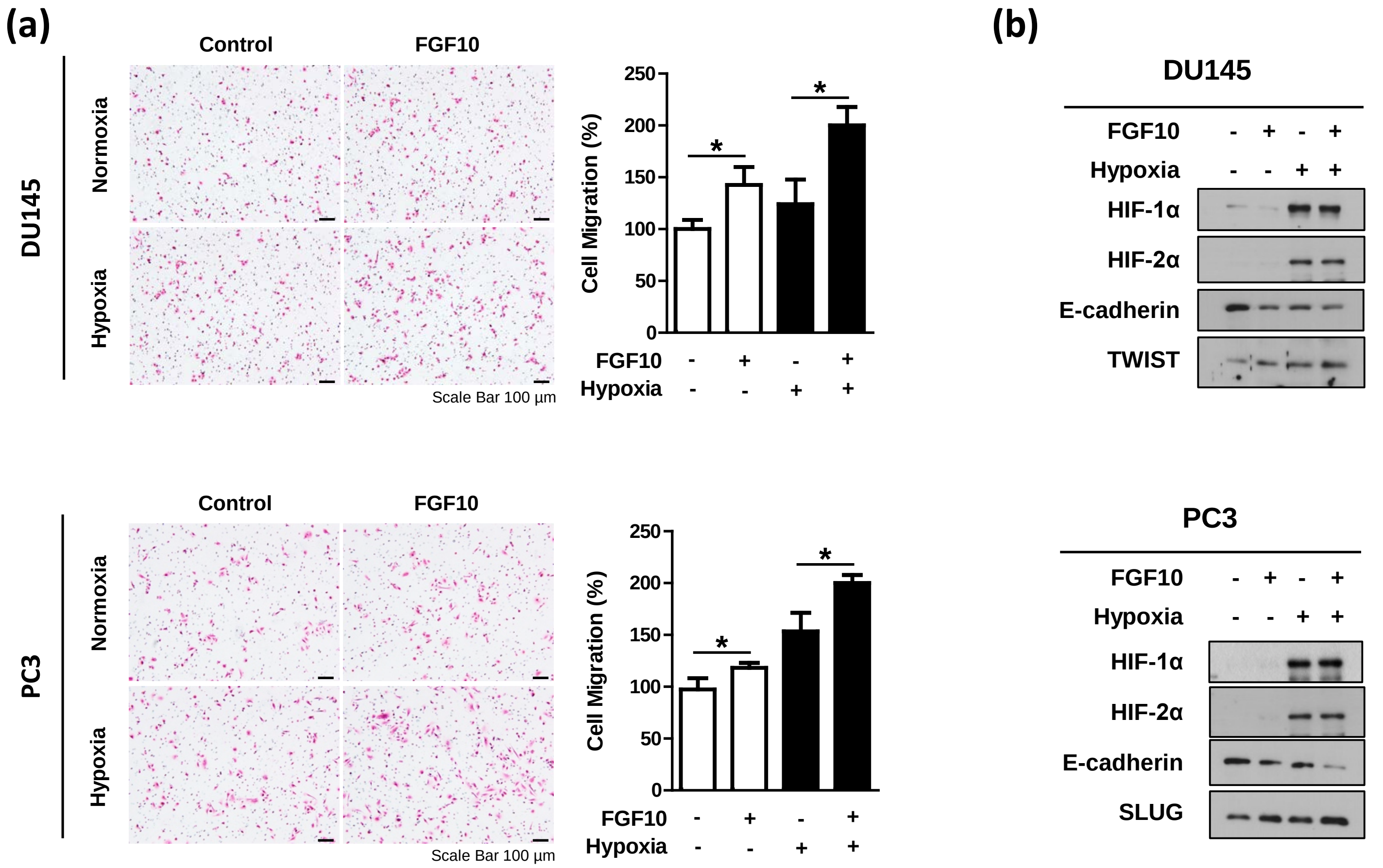

Supplementary Figure 5. DU145 and PC3 cells were serum starved overnight and treated with 50 ng/ml FGF10 for 8 hours, and subjected to cell migration analyses (a). After incubation in normoxic or hypoxic conditions for 24 hours, cells on the lower surface of the membrane were stained with hematoxylin and eosin, and photographed under an optical microscope (left panel). Cells were counted and migrated cell numbers are presented as the means + SD. respectively (right panel). DU145 and PC3 cells were serum starved overnight and treated with 50 ng/ml FGF10 for 8 hours , and incubated under normoxic or hypoxic conditions for 24 hours. Cells were subjected to immunoblotting with the indicated antibodies (b). All experiments were performed three times and results are expressed as bars representing the means + SD. * denotes $p < 0.05$ between the indicated groups, respectively.

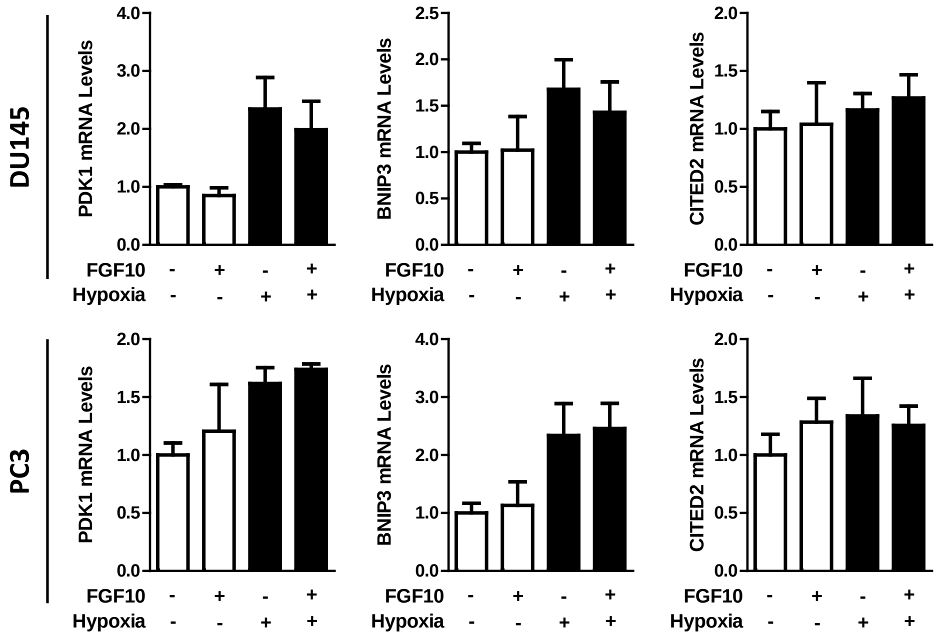

Supplementary Figure 6. DU145 and PC3 cells were serum starved overnight and treated with 50 ng/ml FGF10 for 8 hours, and incubated under normoxic or hypoxic conditions for 24 hours. The mRNA levels of PDK1, BNIP3, and CITED2 were quantified by RT-qPCR. All experiments were performed three times and results are expressed as bars representing the means + SD.

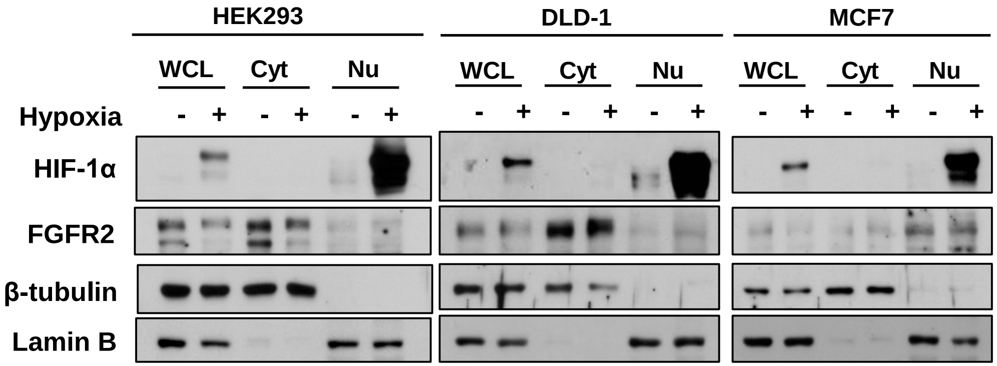

Supplementary Figure 7. Subcellular localization of FGFR2 in other cell lines. HEK293, DLD-1 and MCF-7 cells were incubated in hypoxia for 8 hours, cytosolic fractions and nucleus fractions were obtained by subcellular fractionation. These fractions were subjected to immunoblotting with the indicated antibodies.
